# Nocturnal awakenings of Brazilian immigrants in Massachusetts

**DOI:** 10.5935/1984-0063.20200040

**Published:** 2021

**Authors:** Talita Monsores Paixão, Liliane Reis Teixeira, Eliana Napoleão Cozendey-Silva, Carlos Eduardo Gomes Siqueira

**Affiliations:** 1 Oswaldo Cruz Foundation, National School of Public Health Sergio Arouca - Rio de Janeiro - RJ - Brazil.; 2 University of Massachusetts Boston, School for the Environment - Boston - MA - United States.

**Keywords:** Brazilian Immigrants, Work, Sleep, Sleep Wake Disorders, Sleep Apnea Syndrome, Sleepiness

## Abstract

**Objective:**

The purpose of this study was to assess the relationship between the quantity of jobs and nocturnal awakenings of Brazilians living in Massachusetts.

**Material and Methods:**

We sampled of 48 documented Brazilians around the age of 45.5 years old. 52.1% of them were women. Data gathering occurred for three weeks, using the Pittsburgh Sleep Quality Index. Participants also wore wrist actigraph and ﬁlled sleep/wake diary for a week.

**Results:**

The sleep quality of immigrants with one job (mean=8.58, SD=4.16) is better when compared to immigrants with 2-3 jobs (mean=12.7, SD=3.57) according to the PSQI scores. Immigrants with 2-3 jobs reported dissatisfaction on three components of the scale: sleep duration, sleep efficiency and sleep quality.

**Discussion:**

There is a positive relationship between the quantity of jobs and nocturnal awakenings and between nocturnal awakenings and complaints related to sleep apnea among Brazilians in Massachusetts. The assessment of systemic morbidities associated with sleep pattern changes should be considered in future research.

## INTRODUCTION

The emigration of Brazilians to developed countries has been increasingly prominent since it started some decades ago, and the United States of America is the top hosting country^1^ for Brazilians. This immigrant population consists mainly of young adults entering the informal labor market, and work is the main factor that drives them out of Brazil^2-5^. The state of Massachusetts is one of the main destinations for Brazilian immigrants in the U.S., besides Florida, New Jersey, New York and California^4-6^.

The most employable occupations for Brazilian immigrants are in the food sector, domestic work and civil construction^2,7^. Siqueira^8^ believes that most of these immigrants are informal workers who work in substandard conditions, without work contracts and health insurance, with low wages and long working hours.

It is recognized in the literature that long, uninterrupted working hours, or with few breaks impair physical and mental post-work recovery, even in healthy populations, generating health-related problems such as metabolic changes, gastrointestinal and cardiovascular disorders, altered eating habits, decreased attention and reaction speed, feeling of isolation, fatigue and sleep-related complaints^9-11^.

Besides the most direct occupational factors, especially related to precarious work with long working hours, issues such as low schooling, low income, language barriers, different customs and food, as well as the deportation of family and friends^12^, also appear as aggravating factors to the health of Brazilian immigrants in the U.S.^2,13^. Research shows that Latino immigrants in the U.S. have higher rates of mortality, injuries and illnesses, and greater exposure to occupational risks and workload when compared to the Native American population^6^.

Insomnia^14^ is one of the most common sleep-related complaints, with a prevalence of 10% to 15% depending on the diagnostic criterion adopted and a yearly incidence of 5%. The International Classification of Sleep Disorders^15^ defines insomnia as the complaint of difficulty in initiating or maintaining sleep, associated with daytime consequences (e.g., drowsiness, fatigue and impaired attention) and not attributable to environmental circumstances or inadequate sleeping opportunities. It involves sleep quantity and quality dissatisfaction (nocturnal awakenings and nonrestorative sleep)^16^. Chronic insomnia^15^ occurs when sleep-related complaints persist for at least three months, with a minimum frequency of three times a week. The most common symptom of insomnia is difficulty in maintaining sleep throughout the night (61% frequency), followed by waking up ahead of time (52% frequency) and difficulty falling asleep (38% frequency)^14,17,18^. Insomnia symptoms are often associated with psychosocial factors^19,20^. Hale et al.^21^ affirm that high-risk sleep in immigrants in the U.S. has been associated with chronic psychosocial stressors such as unemployment, low education and low social support.

In a review of the literature on sleep disorders in Latino immigrants in the U.S., among other factors, Baldwin et al.^22^ observed greater insomnia symptoms related primarily to psychological factors in Latino women with a longer life span in the U.S. when compared to other women, shorter sleep duration in non-Mexican Latinos when compared to Caucasians and Mexican Latinos, and excessive daytime sleepiness associated with worse mental health conditions in immigrants.

However, we found no evidence on occupational factors related to emigration and sleep complaints, including insomnia, in a population of Brazilian immigrants. Knowledge about the health-work-sleep disorders among Brazilian immigrants in the U.S. is quite limited^3^.

Published studies^12,21-23^ have focused on comorbidities among Latino immigrants residing in the U.S. However, surveys very rarely include Brazilians. Analyzing Brazilian immigrants separated from Latinos is important due to cultural, social or behavioral differences^7,24^. Thus, this study aimed to contribute to the literature by assessing the relationship between the number of jobs and the nocturnal awakenings of Brazilian immigrants in Massachusetts, U.S.

## MATERIAL AND METHODS

This study used secondary data with restricted access from data collected in Lowell and Framingham, MA, during the winter. A convenience sample of 48 Brazilians in these locations included documented women and men aged 18-65 years. 52.1% of respondents were women.

Participants were recruited through a variety of means, such as brochures distributed in Brazilian companies in both cities, contacts with the Massachusetts Alliance for Portuguese Speakers (MAPS) team, and personal contacts at health fairs and several churches, since the latter are fundamental to the lives of many Brazilian immigrants. The study involved collaboration between the University of Massachusetts campuses in Lowell and Boston and MAPS. MAPS is a non-profit community organization for members of Portuguese-speaking communities which aims at increasing access and removing barriers to health, education and social services through direct services, advocacy, leadership and community development.

Data were collected over three weeks on two successive visits to MAPS offices. In the first visit to the MAPS office in Framingham, MA (day 1), study participants were fully briefed about the study, signed the informed consent form and completed a personal health assessment that included their medical history and the Pittsburgh Sleep Quality Index (PSQI). All documents were in Portuguese. The Portuguese version of the PSQI was validated in a previous study^25^.

The PSQI evaluates subjective sleep quality. It consists of 19 self-applied questions grouped into seven components with weights distributed on a scale of 0 to 3: subjective sleep quality, sleep latency, sleep duration, sleep efficiency, sleep disorders, sleeping medications and daytime sleepiness. The final score of the instrument can range from 0 to 21 points, and the higher the score, the worse the sleep quality. Scores above five points are already indicative of poor sleep quality, and individuals may have great difficulties in at least two components, or moderate difficulties in more than three components^26^.

At the end of the visit, actigraphs were placed on the wrists of the participants, which allowed collecting information on nocturnal awakenings. Participants also received a package of sleep/activity diaries to be completed in the next seven days. One week after the material was delivered, participants went back and returned both.

From day 2 to day 7, participants completed the sleep/activity diaries while constantly wearing the wrist actigraph, which was only removed when bathing. On day 8, participants returned the sleep/activity diaries and the actigraph.

For the next two weeks (between the 15^th^ and the 22^nd^), participants returned to MAPS offices in Lowell and Framingham to receive a summary report of all their results. During this second visit, participants received feedback sheets related to sleep quality and information on sleep hygiene, insomnia and the importance of maintaining sleep quality for optimal health. A nurse who was a sleep specialist carefully explained all results and advised those with abnormalities to schedule appointments with health professionals for specific clinical diagnosis and treatment. In some cases, this nurse wrote specific letters explaining the results of wrist actigraphy to facilitate communication between participants and health care professionals.

We first performed descriptive statistics analysis (distribution of frequencies, means and standard deviations) followed by a normality test (Shapiro-Wilk test). The variables that showed normal distribution were tested using the Student’s t-test and ANOVA. Only the variable nocturnal awakenings did not have a normal distribution and was analyzed by the Mann-Whitney test. The significance level of α=5% was considered in all analyses. The SPSS 23.0 software was used to perform the analysis.

This research was approved by the University of Massachusetts Institutional Review Board (UMass Lowell), per IRD opinion 11-082-PHI-XPD/2011. In Brazil, a study protocol was approved by the Ethics Committee of ENSP/Fiocruz, as per CAAE 3225.1014.7.0000.5240 for access and use of the restricted database.

## RESULTS

The studied population had a mean age of 45.5 years (SD=9.8 years), was documented and had been living on average for 12 years in the U.S. (SD=4.7 years). In total, 53% had completed high school, 66% were married, 71% had up to two children and 80% already had family in the U.S. When they lived in Brazil, 44% were commercial workers, while 33% were cleaning service workers in the U.S. Half of the workers had two jobs, in which 32% worked over 60 hours a week, and 68% worked more than 9 hours a day. Workers with one job had lower level of education (61% had complete high school, 39% had complete middle school and no one had college education) compared to workers with two jobs, but this association was not significant. The body mass index (BMI) showed that 21% were normal weight (18.5-24.9), 46% were overweight (25.0-29.9) and 33% were obese (BMI of 30 or more).

Nocturnal awakenings after the onset of sleep averaged 50.42 minutes (SD=33.39 min), and the longest nocturnal awakening episodes lasted around 32 minutes (SD=17.21 min), without any variation by age and gender (Table 1). Women’s nocturnal awakenings lasted 11.88 minutes longer than men did on some weekdays.

**Table 1:** Mean duration of nocturnal awakening episodes during the week, by gender and age group.

Variables		Monday	Tuesday	Wednesday	Thursday	Friday	Saturday	Sunday
**Gender**								
**Male**	Mean	29.40	40.65	31.11	30.70	32.82	34.95	34.95
	SD	11.68	19.78	16.63	17.54	16.53	17.43	17.43
**Female**	Mean	30.46	27.86	30.27	32.82	28.55	34.21	34.21
	SD	19.25	17.28	12.93	19.84	16.62	12.25	12.25
**Total**	Mean	29.98	33.44	30.65	31.81	30.41	34.56	34.56
	SD	16.09	19.26	14.52	18.58	16.50	14.72	14.72
**Age group**		**Monday**	**Tuesday**	**Wednesday**	**Thursday**	**Friday**	**Saturday**	**Sunday**
**19-42 years**	Mean	31.67	36.40	31.73	27.92	33.27	34.92	36.23
	SD	8.17	13.13	12.54	13.00	11.32	15.09	17.67
**43-48 years**	Mean	27.23	27.69	33.38	30.67	29.27	35.15	32.38
	SD	10.17	19.02	15.53	12.96	18.16	24.04	12.35
**49-68 years**	Mean	30.79	36.25	27.69	35.17	29.29	28.41	34.89
	SD	22.37	22.45	15.28	24.32	18.78	22.45	14.64
**Total**	Mean	29.98	33.44	30.65	31.81	30.41	32.42	34.56
	SD	16.09	19.26	14.52	18.58	16.50	20.80	14.72

SD = Standard deviation.

**Figure 1:**
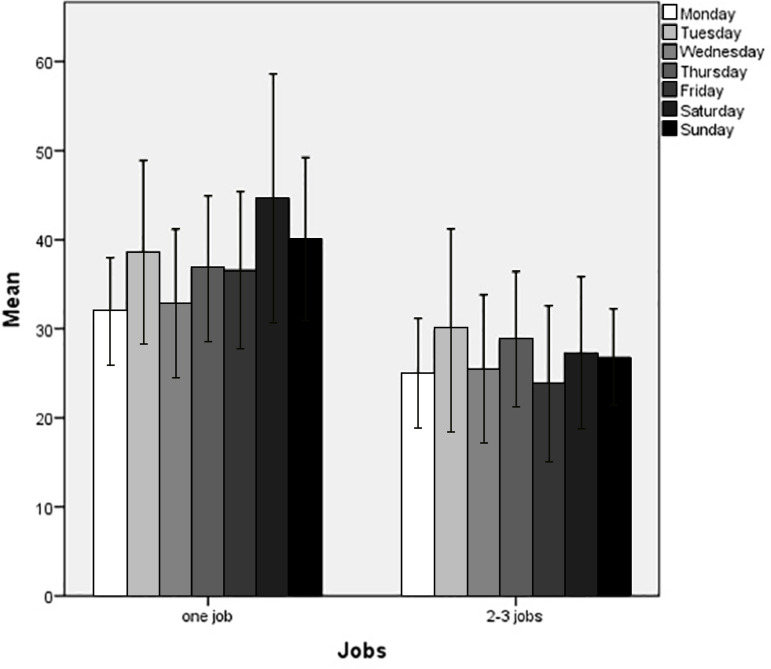
Means of nocturnal awakenings during days of the week, by number of jobs.

The mean nocturnal awakenings by number of jobs fluctuated over the days of the week, with significant differences from Thursday to Sunday (Figure 1). The means are higher for workers with a single job during all days of the week. However, this difference increases by 10-15 minutes from Thursday to Sunday.

Table 2 also shows the higher mean of nocturnal awakenings among immigrants with a single job, on weekdays and weekends, compared to those with 2-3 jobs. Therefore, we found that immigrants with a single job reported longer sleep duration, but less efficiency, due to nocturnal awakenings. Another significant result observed was the duration of nocturnal awakenings higher than 10 minutes for immigrants who reported “stopping breathing” during sleep. After adjusting for gender and age (Table 3), we observed that men of working age (19-50 years old) with a single job had a higher mean duration of nocturnal awakenings on weekends when compared to other groups. Moreover, the higher duration of nocturnal awakenings for immigrants who reported “stopping breathing” during sleep remained significant on weekends, showing that men of working age had a higher mean duration of nocturnal awakenings, when compared to others.

**Table 2:** Mean duration of nocturnal awakenings, for weekdays and weekends, by number of jobs and reported stopping breathing.

Variable		Nocturnal awakenings		Nocturnal awakenings	
		(Weekday)		(Weekend)	
		Mean	SD	Mean	SD
Nº of jobs	1 job	34.83	12.38	41.91**	17.82
	2-3 jobs	26.57	10.23	26.84	9.18
Stopping breathing	Yes	39.89*	14.36	42.27*	17.31
	No	29.72	10.41	30.83	13.43

SD = Standard deviation;

* *p*<0.05;

** *p*<0.01: Mann-Whitney test applied.

**Table 3:** Gender and age adjusted analysis of mean duration of nocturnal awakenings, for weekdays and weekends, by number of jobs and reported stopping breathing.

Variable		Gender	Age (y)	Nocturnal awakenings		Nocturnal awakenings	
				**(Weekday)**		**(Weekday)**	
				**Mean**	**SD**	**Mean**	**SD**
Nº of jobs	1 job	Men	19-50	40.45	15.88	50.64*	16.02
			51-75	30.40	18.10	26.66	8.51
		Women	19-50	31.35	3.67	41.12	24.07
			51-75	29.26	2.61	35.75	5.30
	2-3 jobs	Men	19-50	29.12	7.78	32.28	7.07
			51-75	25.60	0	19.0	1.41
		Women	19-50	26.23	14.29	26.78	8.16
			51-75	23.66	8.30	19.50	12.67
Stopping breathing	Yes	Men	19-50	41.76	16.98	49.50*	17.03
			51-75	0	0	18.00	0
		Women	19-50	40.00	7.35	34.25	8.13
			51-75	28.40	0	32.0	0
	No	Men	19-50	29.70	7.10	30.50	9.50
			51-75	28.46	13.23	25.00	7.71
		Women	19-50	29.10	10.54	34.61	17.41
			51-75	31.20	14.00	29.30	16.44

y = years old; SD = Standard deviation;

* *p*<0.05 Mann-Whitney test applied.

We also observed a significant association (*p*=0.04) between overweight (67%) and obese (33%) immigrants who reported “stopping breathing” during sleep, when compared to normal weight immigrants (none of those reported “stopping breathing” during sleep). There was no significant difference by gender and age.

The mean of nocturnal awakenings related to the question “Does the intensity of snoring cause the partner to leave the room in order to sleep?” is significant for weekdays (39.04 min, SD=14.38) and weekends (44.10 min, SD=16.50). According to Table 4, the mean duration of nocturnal awakenings is 12-15 minutes longer for participants who reported this type of discomfort in the partner. The partners of Brazilian immigrants also reported stopping breathing during sleep, related to the duration of nocturnal awakenings during the week (39.89 min, SD=14.36) and weekend (42.27 min, SD=17.31). When adjusted for gender and age (Table 5), the mean duration of nocturnal awakenings for participants who reported this type of discomfort in the partner remained significant for women and for immigrants of working age on weekends, compared to women and immigrant of working age with partners who did not experience this discomfort. We also observed a borderline association for immigrants of working age on weekdays (*p*=0.058). The analysis of snoring by number of jobs did not show significant results.

**Table 4:** Mean duration of nocturnal awakenings per reported snoring discomfort for partners.

Duration		Snoring disturbs partner	N	Mean	SD
Nocturnal	awakening	Yes	10	39.04	14.38*
(Weekday)		No	16	27.81	8.53
Nocturnal	awakening	Yes	10	44.10	16.50**
(Weekend)		No	21	29.21	9.93

N = Absolute frequency; SD = Standard deviation;

* *p*<0.05;

** *p*<0.01: Mann- Whitney test applied.

**Table 5:** Gender and age adjusted analysis of mean duration of nocturnal awakenings per reported snoring discomfort for partners.

Variable				Nocturnal awakenings		Nocturnal awakenings	
				(Weekday)		(Weekend)	
				Mean	SD	Mean	SD
Snoring disturbs partner	Yes	Gender	Men	40.94	16.67	44.81	18.61
			Women	34.60	7.30	41.26*	1.76
		Age (y)	19-50	40.00	15.56	46.11*	16.14
			51-75	35.20	11.31	26.00	0
	No	Gender	Men	29.34	7.20	30.04	11.46
			Women	26.62	9.68	28.30	8.44
		Age (y)	19-50	28.21	9.68	30.10	10.67
			51-75	26.92	6.04	27.42	8.72

y = years old; SD = Standard deviation;

* *p*<0.05: Mann-Whitney test applied.

The quality of sleep of immigrants with a single job (mean of 8.58, SD=4.16) is better compared to immigrants with 2-3 jobs (mean of 12.7, SD=3.57), according to the PSQI. Immigrants with 2-3 jobs reported dissatisfaction on three components of the scale, namely, sleep duration, sleep efficiency and sleep quality.

The sleep duration reported by immigrants with 2-3 jobs was less than 7 hours a day, while immigrants with only a single job reported sleep duration greater than 7 hours a day. Sleep efficiency (percentage of total sleep time while lying down) for immigrants with 2-3 jobs was over 84%, unlike immigrants with only a single job (less than 84%). In total, 85% of immigrants with 2-3 jobs reported poor or very poor self-reported sleep quality, while 73.7% of those with a single job reported good or very good sleep quality.

In total, 22.9% of immigrants reported taking sleeping pills. When analyzing the association between the use of medications and the duration of nocturnal awakenings, we found a significant association during weekdays, where the mean duration of nocturnal awakenings for those who did not take medication was 27 min (SD=8.25 min), while it was 49.2 min (SD=6.79 min) for immigrants taking medication. The use of melatonin was reported by one immigrant, whose mean of nocturnal awakenings fell to 19.4 min.

## DISCUSSION

Poor sleep quality has a complex relationship with comorbid conditions such as stress, overweight/obesity, anxiety and depression, for example^24,27-30^. Our findings found a positive association between number of jobs and nocturnal awakenings from Thursday to Sunday in Brazilian immigrants in Massachusetts.

Immigrants with a single job had a longer nocturnal awakening, and an increase in these awakenings on weekends was due to social activities, especially drinking. As for the relationship with the number of jobs, for those workers who had a single job^31,32^, the lack of satisfaction with the job, the self-imposed demand to be efficient at work and the fear of losing the job can explain the higher mean of nocturnal awakenings in these individuals. Moreover, the fear and stress generated by the possibility of losing the only job may explain the longer mean duration of nocturnal awakenings in immigrants of working age, who usually have greater concern about the monthly contribution to family earnings, since factors such as anxiety and stress are related to sleep disorders^33^. It may also be related to the higher mean duration of nocturnal awakenings associated with apnea-hypopnea episodes (report of “stopping breathing” during sleep) and snoring intensity in this group, discussed further below.

On the other hand, immigrants with 2-3 jobs, although with mean weekly working hours similar to immigrants with a single job (49.7h and 49.1h, respectively), had to travel more often, besides the probable fragile relationships. In other words, exhaustion resulting from the commitment to two or more jobs and the perception that their financial situation was better in the U.S. than in Brazil, even if the working conditions were worse^23^ or required greater physical effort^27^, could act as inducers for the consolidated sleep period.

The association between snoring and nocturnal awakenings has been relatively well-established^34^ in recent decades. Snoring is an audible condition due to the vibration of soft tissues in the nasopharynx and oropharynx, due to the passage of air during sleep^35^. While snoring is a common and harmless occurrence in many people, it can also appear as a clinical manifestation or warning sign of sleep disorders and comorbidities^29^.

Data from the literature confirm that airways become more vulnerable^36^ during sleep. Bosi et al.^36^ conducted a systematic review of anatomical and non-anatomical factors in patients with obstructive sleep apnea and hypopnea syndrome. They found that both the anatomical factors that predispose upper airway obstruction (UAO) during sleep (e.g., anatomical features and contribution to the narrowing of the upper airways) and neuromuscular factors during sleep (progressive reduction of neuromuscular activity that modulates the excitation, from NREM sleep to the REM sleep stage), are related to restricted airflow or upper airways collapse, and are the main factors for hypopnea and obstructive sleep apnea. In turn, these events are directly associated with nocturnal awakening and sleep fragmentation, contributing to fatigue, daytime drowsiness, reduced professional performance and risk of occupational accidents.

The snoring intensity and apnea-hypopnea episodes can be exacerbated by the relaxation of upper airway structures caused by the use of alcohol or sleep-inducing drugs, given that the neuromuscular compensation factors depend on the waking-sleep state^36,37^. Moreover, the association between BMI and apnea-hypopnea episodes is widely discussed in literature, including in immigrant populations. Studies show^38-40^ that increased body weight, especially obesity, and increased neck circumference are associated with increased episodes of apnea-hypopnea during sleep. This exacerbation could explain the means of snoring intensity (39.9 min, SD=14.4 min for weekdays, and 42.3 min, SD=17.3 min for weekends), the mean duration of nocturnal awakenings (> 10 min) for immigrants who reported “stopping breathing”, the longest mean duration (49.2 min; SD=6.79 min) of nocturnal awakenings for immigrants who took medication compared to those who did not (27 min; SD=8.25 min), and the higher frequencies of overweight and obese immigrants who reported “stopping breathing” during sleep, compared to normal weight immigrants. Moreover, some sleep-inducing pills (e.g., longer-acting medications, indicated for sleep maintenance) alter the sleep architecture in such a way that the N2 of NREM sleep is longer than normal, which can increase sleep time, without, however, ensuring restful sleep^15,36,37,41^.

Concerning the Pittsburgh Sleep Quality Index and number of jobs, the PSQI showed difficulties for immigrants with more than a single job in three components of the scale. In essence, they had shorter sleep duration, lower sleep efficiency and poor or very poor sleep quality, which can be explained by the reduction in time to sleep as a result of increased number of hours worked, similar to that observed by Marucci-Wellman et al.^42^.

The authors who developed the PSQI^26^ believe that sleep quality is an important clinical construct for two reasons. The first is because sleep quality-related complaints (such as difficulty falling asleep or maintaining sleep) are common, and the second is because poor sleep quality can be a symptom of different sleep disorders and other comorbidities. Sleep duration is one of the main components in assessing sleep quality. It is associated with different health problems and may be directly associated with mortality. Findings similar to shorter sleep duration observed in this study for immigrants with more than a single job compared to immigrants with a single job were also observed in other studies^42,43^.

Basner et al.^43^ observed a shorter sleep duration of 47 minutes for workers in multiple jobs. Marucci-Wellman et al.^44^ showed that permanent or temporary workers with more than a single job also showed a 45-minute reduction in sleep duration on weekdays and 62-minutes on weekends, compared to those with a single fixed job or who were unemployed.

The worst sleep quality observed for immigrants with more than a single job in this study is of concern, as those workers may be subject to chronic and acute sleep deprivation due to reduced time to sleep and greater risk of accidents. They may also be subject to systemic fatigue and morbidities and low sleep efficiency^42,43^.

The use of sleeping pills, in general, assists the initiation of sleep, reduces nocturnal awakenings, and increases the mean duration of sleep, but can cause tolerance due to prolonged use. Furthermore, there is no consistency in improving the quality or duration of sleep and reducing nocturnal awakenings^15,37,41^.

This study has some limitations. First, it cannot be said that the findings discussed here are generalizable to other Brazilian immigrants in Massachusetts since the sampling was not random and due to the sample size. However, as there are few studies that assess the associations between sleep and health of Brazilian immigrants, this was an exploratory study that sought to generate hypotheses for future studies. Selection and memory biases may have likely occurred due to the recruitment sites and the subjective nature of some questions. While recruitment occurred in different cities and locations, the most acculturated Brazilians in American Society may not have been included in our sample because they would not visit these locations as often, or perhaps never. Furthermore, because immigrant populations in the U.S. often have multiple jobs, many in this situation may not have had time to participate in the study. Nevertheless, most U.S. immigrants do not have health insurance and may have taken advantage of the surveys as a way to get more information about their health status. Another limitation was the fact that data was collected in the morning, which reduced the participation of immigrants who worked at night.

Also, the Healthy Immigrant Effect, which is the tendency of immigrants to be healthier than the native-born population, especially recent immigrants (up to five years in the host country) and younger ones, is described in the literature as an important feature to be observed in immigrant populations^45^. Yet, since many immigrants in this study had a long time of residence in the U.S. (mean of 12 years), and we did not have participants with less than 5 years of residence in the U.S., the associations that sought to investigate the Healthy Immigrant Effect were not significant.

However, this study can contribute to not only make demands visible but also articulate support strategies for Brazilian immigrants in Massachusetts. Furthermore, its contribution to expand the horizon of health care professionals concerning changes in sleep patterns, the likely increased exposure to occupational risks and the plausible warning sign of comorbidities are made visible.

## CONCLUSION

Our findings identified a relationship between the number of jobs and the nocturnal awakenings of Brazilian immigrants in Massachusetts. Moreover, an association was also found between nocturnal awakenings and sleep apnea-related complaints. However, additional studies are needed to better understand sleep problems in this population and how that affect their health. The assessment of the relationship between systemic morbidities (e.g., metabolic syndrome) and changes in sleep patterns in Brazilian immigrants should be considered in future research.
